# Augmentation vs. switching medications in older patients with treatment-resistant depression: clinical moderators that matter

**DOI:** 10.1192/j.eurpsy.2025.474

**Published:** 2025-08-26

**Authors:** H. K. Kim, J. F. Karp, H. Lavretsky, D. M. Blumberger, P. J. Brown, A. J. Flint, E. Lenard, P. Miller, C. F. Reynolds, S. P. Roose, E. J. Lenze, B. H. Mulsant

**Affiliations:** 1 Psychiatry, University of Toronto; 2 Centre for Addiction and Mental Health, Toronto, Canada; 3 Psychiatry, University of Arizona, Tucson; 4 Psychiatry, University of California, Los Angeles, Los Angeles, United States; 5 Psychiatry, Centre for Addiction and Mental Health, Toronto, Canada; 6 Clinical Medical Psychology in Psychiatry, Columbia University, New York, United States; 7 Psychiatry, University Health Network, Toronto, Canada; 8 Psychiatry; 9 Informatics, Data Science & Biostatistics, Washington University in St. Louis, St. Louis; 10 Psychiatry, University of Pittsburgh Medical Centre, Pittsburgh; 11 Psychiatry, Columbia University, New York, United States

## Abstract

**Introduction:**

Older adults with treatment-resistant depression (TRD) can be treated with augmentation or switched to a different drug.

**Objectives:**

We aimed to identify factors that moderate the effectiveness of these strategies on treatment outcomes to guide the selection of the optimal strategy for each patient.

**Methods:**

We analyzed data from 742 older adults with TRD in the Outcomes of Treatment-Resistant Depression in Older Adults (OPTIMUM) clinical trial. All participants were randomized to one of two treatment strategies, which were augmentation with aripiprazole, bupropion, or lithium; or switching to bupropion or nortriptyline. Treatment outcomes were change in MADRS scores and remission after 10 weeks. Age, burden of comorbid physical illness, number of adequate previous antidepressant trials, presence of executive cognitive impairment, and clinically relevant comorbid anxiety were examined as potential moderators of the effect of the two treatment strategies (augmentation vs. switching) on treatment outcomes.

**Results:**

Overall, augmentation produced more improvement in MADRS scores and produced a higher rate of remission than switching. For change in MADRS scores after 10 weeks of treatment, the number of adequate previous antidepressant trials was the only significant moderator of the superiority of augmentation over switching (b = -1.6, t = -2.1, p = 0.033, 95%CI [-3.0,-0.1]). There were no significant moderators for remission.

**Conclusions:**

Older patients with TRD with less than three previous antidepressant trials benefit more from augmentation than from switching. Future studies validating this finding with different drugs in more diverse samples can facilitate their application in real world settings.

**Disclosure of Interest:**

H. Kim Grant / Research support from: Dr. Kim reports grant support from the PSI foundation (R23-21). She is supported by the Canadian Institutes of Health Research (CIHR) and the Temerty Faculty of Medicine (Chisholm Memorial Fellowship)., J. Karp: None Declared, H. Lavretsky Grant / Research support from: Dr. Lavretsky received support from grants (K24 AT009198, R01 AT008383, and R01 MH114981) from the NIH., D. Blumberger Grant / Research support from: Dr. Blumberger reports grants from Canadian Institutes of Health Research (CIHR) and the Temerty family through the Centre for Addiction and Mental Health (CAMH) Foundation during the conduct of the study; nonfinancial support from Magventure (in-kind equipment support for investigator-initiated research); grants from Brainsway (principal investigator of an investigator-initiated study and site principal investigator for sponsored clinical trials), National Institutes of Health (NIH), Brain Canada Foundation, Campbell Family Research Institute, and Patient-Centered Outcomes Research Institute outside the submitted work; received medication supplies for an investigator-initiated trial from Indivior; and has participated in advisory boards for Janssen and Welcony., P. Brown Grant / Research support from: Dr. Brown received additional support from the National Institute of Mental Health OPTIMUM NEURO grant (5R01MH114980)., A. Flint Grant / Research support from: Dr. Flint has received grant support from the US National Institutes of Health, the Patient-Centered Outcomes Research Institute, the Canadian Institutes of Health Research, Brain Canada, the Ontario Brain Institute, and Alzheimer’s Association., E. Lenard: None Declared, P. Miller: None Declared, C. Reynolds Shareolder of: Dr. Reynolds receives payment from the American Association of Geriatric Psychiatry as Editor-in-Chief of the American Journal of Geriatric Psychiatry and royalty income for intellectual property as co-inventor of the Pittsburgh Sleep Quality Index., S. Roose: None Declared, E. Lenze Grant / Research support from: Dr. Lenze received additional support from the Taylor Family Institute for Innovative Psychiatric Research at Washington University School of Medicine, as well as the Washington University Institute of Clinical and Translational Sciences grant (UL1TR002345) from the National Center for Advancing Translational Sciences of the National Institutes of Health (NIH)., B. Mulsant Grant / Research support from: Dr. Mulsant received additional support from the Labatt Family Chair in Biology of Depression in Late-Life Adults at the University of Toronto. He holds and receives support from the Labatt Family Chair in Biology of Depression in Late-Life Adults at the University of Toronto. He currently receives or has received during the past three years research support from Brain Canada, the CAMH Foundation, the Canadian Institutes of Health Research, and the US National Institutes of Health (NIH); Capital Solution Design LLC (software used in a study funded by CAMH Foundation), and HAPPYneuron (software used in a study funded by Brain Canada).

